# A new primate from the late Eocene of Vietnam illuminates unexpected strepsirrhine diversity and evolution in Southeast Asia

**DOI:** 10.1038/s41598-019-56255-8

**Published:** 2019-12-27

**Authors:** Olivier Chavasseau, Yaowalak Chaimanee, Stéphane Ducrocq, Vincent Lazzari, Phan Dong Pha, Mana Rugbumrung, Jérôme Surault, Dang Minh Tuan, Jean-Jacques Jaeger

**Affiliations:** 1 PALEVOPRIM UMR CNRS 7262, University of Poitiers, 6 rue Michel Brunet, 86073 Cedex, Poitiers France; 2 Institute of Marine Geology and Geophysics, Vietnam Academy of Science and Technology Building A27, 18 Hoang Quoc Viet Street, Cau Giay District, Hanoi, Vietnam; 3 Graduate University of Science and Technology, Building A21, 18 Hoang Quoc Viet Street, Cau Giay District, Hanoi, Vietnam; 4 Department of Mineral Resources, Rama VI Road, 10400 Bangkok, Thailand

**Keywords:** Palaeontology, Biodiversity

## Abstract

Sivaladapidae is a poorly known Asian strepsirrhine family originally discovered in Miocene sediments of the Indian subcontinent. Subsequent research has considerably increased the diversity, temporal range, and geographical distribution of this group, now documented from China, Thailand, Myanmar, Pakistan, and India and whose earliest representatives date back to the Middle Eocene. We present here a new taxon of sivaladapid from the Na Duong coal mine in the Latest Middle Eocene-Late Eocene of Vietnam. It represents the first Eocene primate from Vietnam and the first medium-sized mammal recovered from this locality, thus documenting a completely new part of the Na Duong paleobiodiversity. This taxon is the largest sivaladapid ever found with an estimated body weight of 5.3 kg and it represents a new subfamily of sivaladapids in exhibiting a very peculiar combination of dental features yet unknown in the fossil record of the family (e.g., retention of four premolars, high-crowned molars with accentuated bunodonty and extreme crest reduction). Besides documenting a complete new part of sivaladapid evolution, its primitive dental formula and derived features shared with the Early Eocene Asiadapidae reinforce the hypothesis of a basal branching of sivaladapids among strepsirrhines.

## Introduction

**Figure 1:**
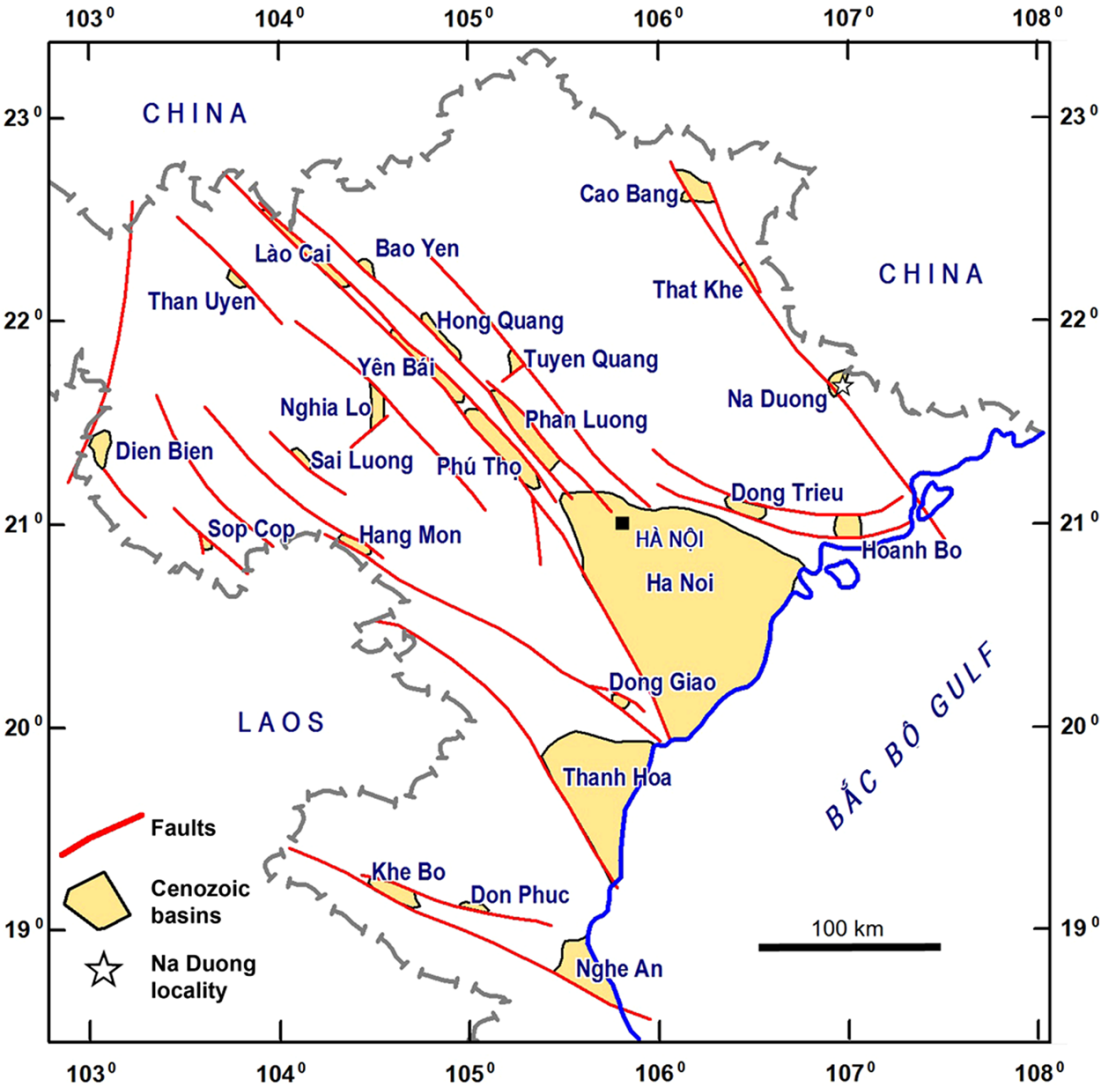
Map of northern Vietnam showing the Cenozoic tectonic basins and the localization of the Na Duong locality. This map was generated with MapInfo Pro 12 (https://www.infosig.net/).

The basin of Na Duong, in Lang Son province of northern Vietnam, is a pull-apart basin along the Cao Bang-Tien Yen transform fault zone (Fig. 1), which is filled by alternated clays and lignite layers deposited in a lacustrine environment^1–3^. The coal mine of Na Duong has recently yielded a fauna of large mammals^1,4,5^ comprising four anthracotheres belonging to the genera *Bothriogenys*, *Anthracokeryx, Elomeryx*^5^, and the rhinocerotid *Epiaceratherium*.

The chronological setting of the Na Duong basin has been mostly discussed based on biochronological data. While previous age estimates placed Na Duong either in the Miocene^3^ or the Oligocene^1^, the basin has since been recognized as Eocene in age based on mammalian biochronology with a proposed Late Bartonian-Priabonian age interval (~39–35 Ma)^4^. More recently, the age of the Na Duong fauna has been restricted to a latest Bartonian-early Priabonian interval (~38–36 Ma) based on biochronological data provided by anthracotheres^5^. Thus, Na Duong offers a time interval yet unknown in Southeast Asia and China being older than the locality of Krabi (Thailand) and younger than the Pondaung Formation localities (Myanmar) and the basins from the Yunnan and Guangxi provinces (China). Although the Na Duong fauna compares well with those from the Middle and Late Eocene of Myanmar, Thailand, and China^5^, all mammals described from Na Duong are new species, which point out the interest of this locality for documenting the paleobiodiversity of the poorly-sampled 38–36 Ma interval. Recently, *Anthracokeryx naduongensis* has been identified from the Youganwo Formation in Guandong province^6^, China, strengthening the faunal affinities between Southeast Asia and Southern China during the Middle and late Eocene.

Until now, only large-bodied cetartiodatyls and perissodactyls are documented from Na Duong. We describe here a new middle-sized primate, which unveils a completely new part of the mammalian paleobiodiversity of Na Duong and of the evolution of Asian primates during the Paleogene.

## Results

### Systematic paleontology

Order Primates Linnaeus, 1758

Suborder Strepsirrhini Geoffroy, 1812

Family Sivaladapidae Thomas and Verma, 1979

Subfamily Anthradapinae subfam. nov.

### Type and only genus

*Anthradapis* gen. nov.

### Diagnosis

Large-sized sivaladapid (body weight >5 kg) possessing a high-crowned m1 with thin enamel, marked exodaenodonty, accentuated bunodonty, metaconid in line with the protoconid, accessory cuspules (centroconid and metastylid), reduced crests (including a weak mesiodistally-oriented cristid obliqua) and absent hypocristid, closed trigonid with large, mesially-pointing and medially-positioned paraconid, deep talonid basin almost closed lingually and open buccally and distally, closely-spaced entoconid and hypoconulid with distally shifted entoconid and small hypoconulid near the midline of the tooth; presence of a single-rooted p1; non-molarized premolars (single-cusped p1 and p2; bicuspid p3-p4 with small hypoconid) with four crests extending from the protoconid including a mesiodistally oriented preprotocristid; premolar row with gentle increase in size from p1 to p4; strongly molarized, elongated, three-lobed bunodont dp4 with reduced crests including weak mesiodistally-oriented cristid obliqua, large and deep talonid basin that is open buccally and notched lingually, well-developed mesial lobe with long and arcuate preprotocristid, wide and open trigonid basin, weak distal shift of the metaconid relative to the protoconid, and accessory cuspules; large deciduous canine with high, vertically implanted, laterally compressed and lingually grooved crown with oval cross-section, large and long-rooted permanent canine with triangular crown tip in lateral perspective and cross-section; proportionally short toothrow and deep mandibular corpus; sequence of eruption of the premolars: p2-p1-p3-p4.

Differs from all known sivaladapids by the retention of a p1, p2 with four crests emanating from the protoconid, a square, bunodont and exodaenodont m1 with extreme crest reduction, open talonid basin with accessory cuspules, and lack of buccal cingulid, a more laterally compressed canine with a lingual vertical groove. Differs from all sivaladapids except *Paukkaungia* and *Kyitchaungia* in having a distinct mesiodistally-oriented cristid obliqua on m1. Differs from all Paleogene sivaladapids (Hoanghoniinae and Wailekiinae^7^) in having a p2 nearly as high and only slightly smaller than p3. Differs from all Paleogene sivaladapids except *Paukkaungia* and *Yunnanadapis* in having a metaconid in line with the protoconid on m1. Further differs from the Neogene sivaladapids (Sivaladapinae) by the presence of four crests connected to the protoconid on lower premolars including a distal postprotocristid connected to the cristid obliqua, the non-molarized and non-enlarged p4, the distally increasing height of the premolars, and the two-rooted non-caniniform p2.

Genus *Anthradapis* gen. nov.

### Diagnosis

As for subfamily.

*Anthradapis vietnamensis* sp. nov.

### Etymology

Genus name derives from the Greek ‘anthrax’ (coal) and from the suffix ‘adapis’ which refers to the adapoid affinities of this primate. The species name derives from the name of the country of discovery.

### Diagnosis

As for subfamily.

### Holotype

**Figure 2:**
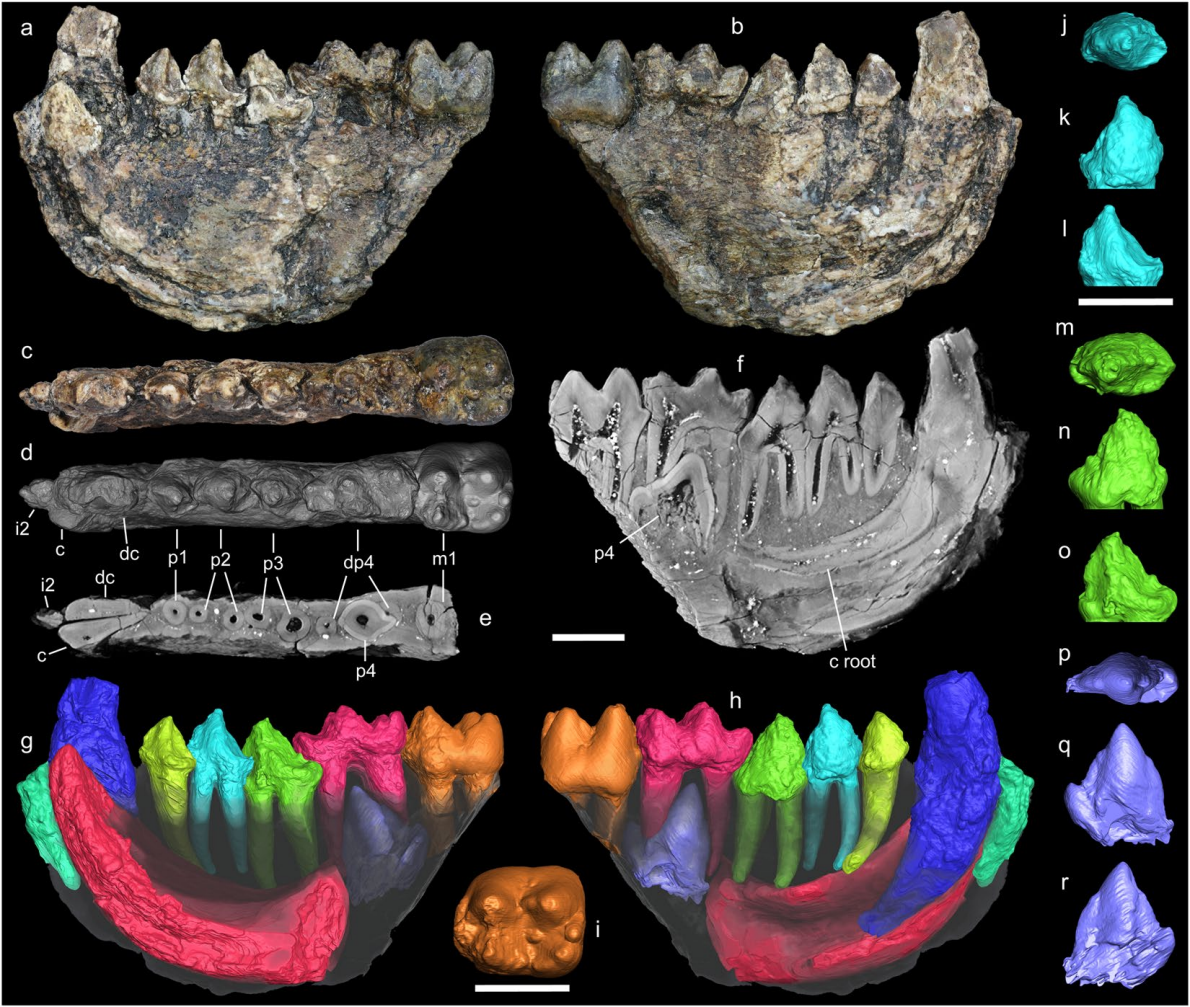
Holotype mandible of *Anthradapis vietnamensis* (ND-2015-12-7) represented in lingual view (**A**), buccal view (**B**), occlusal view (**C**), occlusal 3D rendering (**D**), horizontal virtual section showing roots (**E**), longitudinal virtual section showing roots and p4 germ (**F**), 3D rendering showing manually segmented crowns and/or roots of i2-m1 and p4 germ (**G**,**H**). I-R: 3D rendering of segmented teeth: m1 in occlusal view (**I**); p2 in occlusal (**J**), buccal (**K**) and lingual (**L**) views; p3 in occlusal (**M**), buccal (**N**) and lingual (**O**) views; virtually extracted p4 in occlusal (**P**), buccal (**Q**) and lingual (**R**) views. Scale bars: 5 mm. All virtual sections and 3D renderings were produced with Aviso 7.0 (Visualization Sciences Group, http://www.vsg3d.com/).

ND-2015-12-7 right hemi-mandible of a juvenile individual preserving i2 root, dc, c, p1-p3, dp4, p4 in its socket, and m1 (Fig. 2). The holotype is the only-known specimen of the species.

### Locality and horizon

Na Duong coal mine, latest Middle to middle Late Eocene^5^, Na Duong Fm., lower coal seam, 10 m below the fossiliferous main coal seam^1,5^.

## Description

The mandibular corpus is preserved from the level of i2 to that of m1. It is deep, proportionally slender and straight, showing no significant buccolingual torsion. The tooth row is rectilinear along the mesiodistal axis of the mandible. The surface of the teeth has been partly affected by chemical weathering and taphonomic abrasion. However, most of the original occlusal pattern of the tooth crowns is visible and dietary wear is noticeable on several teeth. This specimen is a juvenile based on the presence of a deciduous canine and a dp4.

The deciduous canine (dc) possesses a vertically implanted and large-sized crown with marked buccolingual compression and oval cross-section. Despite a large apical wear facet, this tooth is high-crowned and much higher than the rest of the tooth row. There is a deep vertical groove on the lingual side of the crown, and no cingulid or talonid. The root is massive, slightly curved distally, and it ends just above the bottom of the corpus below p1. An incisor root, likely representing i2, is located just in front of the dc and shows an almost vertical implantation.

The canine is located just below and lingual to the deciduous canine. This tooth was erupting at the time of the death of the individual, judging its unworn crown much lower than that of the deciduous canine. CT–scan analysis reveals that the massive, long and curved canine root is still inserted deeply in the jaw as far back as the level of p3 and is open distally. The part of the canine crown that has erupted, is triangular in lingual aspect and possesses a triangular transverse section. A shallow vertical groove is present on the mesiolingual side of the crown.

The tooth row is short with only a ~1 mm diastema between dc and p1. The premolar crowns show marked crowding in lateral perspective. The occlusal pattern of the premolars is strikingly similar, being rather constant from p1 to p4. Light occlusal dietary wear is present at the apex of the protoconids of p1-p2. The p1 bears a large single root and only one cusp. It presents a sub-oval occlusal outline with its long axis oblique relative to the longitudinal axis of the tooth row. The p1 crown is asymmetrical in several respects: (1) the buccal wall is regularly convex while the lingual wall presents concavities; (2) the base of the crown is expanded distolingually; and (3) in lateral view, the crown displays a triangular outline with a distal side longer than the mesial one due to a mesial position of the protoconid. This cusp is tall and four crests originate from it: a mesiodistally-oriented preprotocristid which bears no paraconid, a distolingual postprotocristid, a faint distobuccal postprotocristid and a distal postprotocristid, which starts, unlike the others crests, from below the tip of the protoconid. The distobuccal and distolingual postprotocristids delimit a distal basin separated into two parts by the distal postprotocristid. The distolingual cingulid is strong but interrupted at the level of the protoconid.

The two-rooted p2 is larger than p1, and proportionally more elongated than it, bearing no lingual expansion. It differs from the p1 in having a taller crown, a more acute protoconid tip, a stronger development of the distolingual and distobuccal postprotocristids, a small talonid with an incipient hypoconid, a larger distal basin, a slightly lingually-curved preprotocristid, and in lacking a lingual cingulid. The mesial root is very slightly shifted buccally relative to the distal root. The p3 is a larger version of p2 with a slightly taller and proportionally wider crown, a more expanded talonid with a small hypoconid and a rounded distocristid enclosing a larger and deeper distal basin. There is no metaconid and there is a slight buccal shift of the mesial root like on the p2.

The p4 germ was virtually extracted from the mandible. The crown is damaged and lacks its distobuccal part. However, enough of p4 is preserved to observe that it is not molarized and structurally similar to p1-p3. It bears a large and tall protoconid, a well-marked and thick preprotocristid which is slightly curved lingually, and a well-developed talonid with a hypoconid larger and taller than on p3. Thick distal and distolingual postprotocristids are visible but there is no distobuccal postprotocristid (probably because of tooth preservation and extraction). The distal postprotocristid reaches the base of the hypoconid where it joins a small cristid obliqua. A rounded postcristid enclosing the talonid basin is present on the distolingual side but it is too fragmentary to observe if an entoconid was present. The presence of p4 in its socket and the strong crowding with partial overlap of the p1-p3 crowns allow us to determinate their sequence of eruption as p2-p1-p3-p4: the talonid of the p1 and and the trigonid of the p3 are positioned partly below the crown of the p2, which had thus erupted first. The deep position of the p3 crown indicates that it most likely erupted after p1. This sequence is corroborated by the fact that p2 presents more apical wear than p1. The dentine visible at the apex of p3 is interpreted as resulting from to a break rather than from dietary wear, the dentine pit having an unusual longitudinal shape and being much lower than the surrounding enamel. The deduced eruption sequence differs from that of most fossil adapoids, which generally show a p2-p4-p3 or p4-(p2-p3) sequence^8,9^. Analyses of tooth dimensions (Table 1) and proportions (Supplementary Table S1) in *Anthradapis* reveal that the size of the permanent premolars increases modestly from p1 to p4. The p4 was likely slightly larger than p3 but shorter than m1.

**Table 1:** Tooth and corpus dimensions (in mm) of *Anthradapis vietnamensis*.

Tooth	MD	BL	H	MD:BL	Corpus depth	Corpus breadth
c	>4.39	>2.12	>4.74	2.07	—	
dc	5.58	2.92	>7.11	1.91	12.61	
p1	3.80	3.24	3.97	1.17	14.46	
p2	4.59	3.44	5.06	1.33	14.91	4.52
p3	5.69	3.63	5.41	1.57	14.09	
dp4	8	4.26	4.28	1.88	14.88	
p4	>5.95	>2.80	≈6.09	—	—	
m1	7.09	6.14	6.28	1.15	—	5.49

MD: mesiodistal length. BL: buccolingual breadth. H: height of crown.

The dp4 is a double-rooted, elongate and strongly molarized tooth possessing three lobes and five main cusps. This tooth presents apical wear exposing small dentine pits on several cusps and it had erupted before the distinctly less worn p1-p3 and m1. The occlusal pattern is markedly bunodont with all cusps being rounded, especially the buccal ones. The first lobe is the narrowest and bears a low, long and arcuate preprotocristid. A large, deep and lingually open trigonid basin is present. The second lobe bears a protoconid and a metaconid, which are close to each other but separated by a deep valley. The metaconid is more distal than the protoconid, less rounded than the protoconid, and it shows a faint premetacristid and a more distinct postmetacristid. The third lobe is the widest and comprises three peripheralized cusps that delimit a deep and large talonid basin. The hypoconid is located slightly more mesially than the entoconid, which is also smaller and less inflated. A preentocristid bearing a tiny cuspule (preentoconid) is noticeable and, together with the postmetacristid, they partially enclose the talonid basin lingually, with a distinct notch remaining at the junction of the two crests. The talonid basin is mostly open buccally, the cristid obliqua being reduced to a weak and short mesiodistal crest that does not reach the base of the protoconid. A distally pointing hypoconulid is present buccal to the mesiodistal axis of the tooth and lies against the hypoconid. The hypoconulid is smaller than the hypoconid and is connected to the entoconid by a long postentocristid and to the hypoconid by a shorter hypocristid.

The m1 is high-crowned, markedly bunodont and exodaenodont. The trigonid is rather short mesiodistally and exhibits a large, low, centrally positioned and mesially salient paraconid. The metaconid and protoconid are equal in size, transversely in line with each other, and connected by a faint and deeply notched crest. The protoconid and the metaconid display0 very weak preprotocristid and premetacristid respectively. The talonid shows a very large and deep basin delimited by a large hypoconid slightly lower than the other main cusps, a large and distally shifted entoconid and a smaller hypoconulid near the midline of the tooth that is situated closely to the entoconid. There is neither a postentocristid nor a hypocristid. Instead, the hypoconulid is separated from the entoconid by a deep notch and from the hypoconid by a deeper and wider groove, the talonid basin being open distally as a consequence. As on the dp4, the cristid obliqua is mesiodistally-oriented and very reduced so that it does not reach the base of the protoconid, which buccally opens the talonid basin. A weak postmetacristid that ends with a small metastylid and a weak preentocristid are present and are separated by a distinct notch that slightly opens the talonid basin lingually. A small accessory cuspule (‘centroconid’) is visible in the middle of the talonid basin. Only the mesial side of the tooth bears a cingulid. The enamel is thin (RET = 6; see Methods) and falls in the range of extant strepsirrhines^10,11^.

## Comparisons

Despite the morphology of its dp4, the dentition of ND-2015-12-7 is unlike that of any Paleogene ungulate (Supplementary Information) and we thus recognize it as a primate owing to the combination of large deciduous canine and a large permanent canine with vertical implantation, short jaw without diastema, proportionally short and deep corpus, and m1 with a broad talonid basin which corresponds to morphological characteristics commonly found in this order. This identification is reinforced by a phylogenetic analysis performed with a datamatrix of eutherian mammals^12^ which reconstructs *Anthradapis* as a primate (Supplementary Information).

*Anthradapis* is attributed to Strepsirrhini because it possesses a double-rooted p2 (single-rooted p2 in Haplorrhines^13^). Haplorhine afiinity is further discarded because *Anthradapis* possesses several features lacking in the major haplorrhine groups of the Eocene: a vertically-oriented large canine and a lack of distal enlargement of the premolars (unlike in omomyids); it has lower molars with rounded cusps, with short and blunted crests (unlike in tarsiids); it retains a p1 and its premolars are nearly aligned with the long axis of the tooth row (unlike in anthropoids^14^). The Amphipithecidae, the most common Eocene Asian anthropoids in Southeast Asia, whose anthropoid status has been debated^15^, markedly differ from *Anthradapis* in their ‘spatulate’ lower premolars with high paraconid and hypoconid, and buccolingually shifted roots, proportionally deeper jaw and m1 with lower crowns, narrower trigonids, and absent paraconid and hypoconulid^16–18^.

Among strepsirrhines, *Anthradapis* differs from the Ekgmowechashalidae^19^ which lack a p1, have a molarized p4, mesially protruding protoconids on c-p3, and buccally positioned cristid obliqua on lower premolars; they also have strong metastylids and/or postmetacristids and widely-spaced entoconid and hypoconulid on m1^19–22^. Adapids are dissimilar from *Anthradapis* in their molarized lower premolars with sharp crests and their molars with oblique and strong cristid obliqua, marked distal shift of the metaconid, and widely-spaced entoconid and hypoconulid. *Anthradapis* is distinguished from Eocene African advanced stem-strepsirrhines and crown strepsirrhines (Azibiidae, Djebelemuridae, *Karanisia*, *Saharagalago*, *Wadilemur*) which have either sectorial premolars or downwardly-sloped anterior dentition, strongly oblique cristid obliqua and marked distal shift of the metaconid on m1^23–26^. The Eocene African caenopithecine adapiforms (*Aframonius*, *Afradapis*, *Masradapis, Namadapis*^27–29^) also differ from *Anthradapis* in their rectangular m1 with sharp crests, oblique cristid obliqua, narrow trigonid that lacks a paraconid, and marked distal shift of the metaconid, their broader p3/p4 with arcuate preprotocristid and more lateral postprotocristids delimiting a larger talonid basin (*Aframonius*, *Masradapis*), and their reduced dental formula (*Afradapis*).

**Figure 3:**
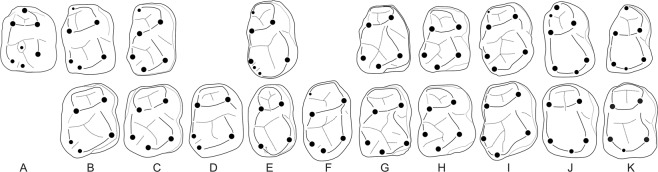
Interpretive drawings comparing the m1 of *Anthradapis* (A) with the m1 (upper row) and m2 (lower row) of other Asian adapoids (**B**–**K**). (**B**–**I**) Paleogene sivaladapids. (**B**) *Hoanghonius stehlini* (m1 of IVPP V10220 (mirrored) after ref. ^48^; m2 of the holotype (University of Uppsala, unnumbered; mirrored) after ref. ^35^). (**C**) *Rencunius zhoui* (m1-m2 of the holotype IVPP 5312 after ref. ^49^). (**D**) *Wailekia orientale* (m2 of the holotype TF 2632 after ref. ^37^). (**E**) *Paukkaungia parva* (m1 NMMP 55 (holotype), m2 NMMP 57 after ref. b^36^). (**F**) *Kyitchaungia takaii* (holotype m2 NMMP 28 after ref. ^36^). (**G**) *Laomaki yunnanensis* (m1 IVPP V 22711 and m2 IVPP V 22712 after ref. ^19^). (**H**) *Yunnanadapis folivorus* (m1 and m2 of the holotype IVPP V 22702 (mirrored) after ref. ^19^). (**I**) *Guangxilemur singsilai* (upper row: m1 or m2 DBC 2171 (mirrored); lower row: m1 or m2 DBC 2170. After ref. ^38^). (**J**–**K**) Asiadapidae (after ref. ^31^). (**J**) *Asiadapis cambayensis* (m1-m2 of the holotype GU 6). (**K**) *Marcgodinotius indicus* (m1 GU 44 and m2 GU 45 (both mirrored)). Teeth not on scale except for molars belonging to the same species for which proportions are respected.

**Figure 4:**
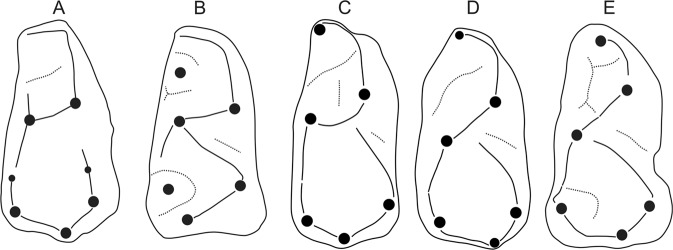
Interpretive drawings comparing the dp4 of *Anthradapis* (**A**) with those of various strepsirrhine primates (**B**–**E**). (**B**) *Sivaladapis nagrii* (LUVP 14505, after Gingerich and Sahni, 1984). (**C**) *Marcgodinotius indicus* (GU 41). (**D**) *Asiadapis cambayensis* (GU 33). (**C**,**D**) after ref. ^31^. (**E**) *Donrussellia lusitanica* (SV3-178, after ref. ^50^). Teeth are not on scale. (**B**,**C** and **E**) have been mirrored.

Most of the Notharctidae differ from *Anthradapis* in their oblique and strong cristid obliqua, widely-spaced entoconid and hypoconulid on m1, and their metaconid on p4^30,31^. However, the Early Eocene Asiadapidae *Asiadapis* and *Marcgodinotius* from India, that were first attributed to the Notharctidae^31^ and subsequently placed in their own family^32,33^, share several characters with *Anthradapis*. Their m1 display a hypoconulid closer to entoconid sometimes with a groove separating these cusps, a buccally-located cristid obliqua with a mostly mesiodistal orientation, a metaconid in line with the protoconid, as well as a large and centrally-located paraconid (Fig. 3). In addition, *Anthradapis* and the asiadapids have a dp4 that displays an elongate and trilobate crown with a narrower mesial lobe bearing a long and markedly lingually-curved preprotocristid, a distally shifted metaconid relative to the protoconid, a wide and lingually open trigonid basin, a large and deep talonid basin and a hypoconulid placed more closely to the hypoconid (*Asiadapis*) (Fig. 4). The dp4 of the basal adapoid *Donrussellia* (Fig. 4E) also resembles that of *Anthradapis* it differs from it mostly in its less elongate mesial lobe associated with a shorter and less curved preprotocrista and by its very oblique cristid obliqua. Hence, the m1 and dp4 features of *Anthradapis* pinpoint to adapoid affinities, and more specifically Asian adapoid affinities. Detailed comparisons (Supplementary Tables S2 and S3) reinforce this interpretation by demonstrating that 41 features of *Anthradapis* can be found among Asian adapoids (including 33 common features). Nevertheless, *Anthradapis* cannot be satisfyingly attributed to the Asiadapidae since there is an important morphological gap with these much more plesiomorphic adapoids (e.g., more simple premolar structure with mesially-projecting mesial walls, low molar talonid).

**Figure 5:**
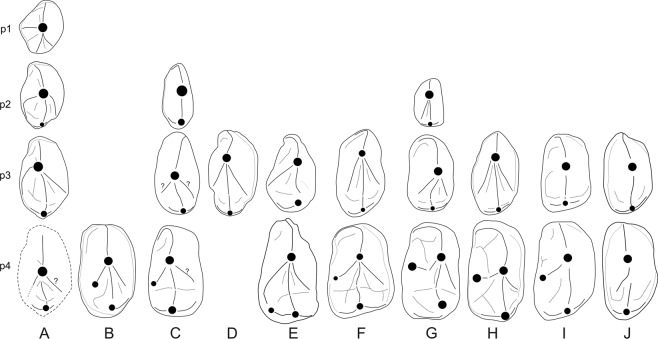
Interpretive drawings comparing the premolars of *Anthradapis* (**A**) with those of other Asian adapoids (**B**–**J**). (**B**–**H**) Paleogene sivaladapids. (**B**) *Rencunius zhoui* (mirrored p4 of the holotype IVPP 5312. After ref. ^49^). (**C**) *Hoanghonius stehlini* (p2-p4 of IVPP V10220 (mirrored). After ref. ^48^). (**D**) *Guangxilemur singsilai* (p3 DBC 2169 (mirrored). After ref. ^38^). (**E**) *Paukkaungia parva* (p3 NMMP 54, p4 NMMP 56 (both mirrored). After ref. ^36^). (**F**) *Laomaki yunnanensis* (p3 IVPP V 22709 and p4 IVPP V 22710. After ref. ^19^). (**G**) *Yunnanadapis folivorus* (p2-p4 of the holotype IVPP V 22702 (mirrored). After ref. ^19^). (**H**) *Yunnanadapis imperator* (p3 IVPP V 22707 (mirrored) and p4 IVPP V 22706 (holotype). After ref. ^19^). (**I**,**J**) Asiadapidae (after ref. ^31^). (**I**) *Asiadapis cambayensis* (mirrored p3-p4 of GU 745). (**J**) *Marcgodinotius indicus* (p3 GU 703 and p4 GU 40). Teeth not on scale except for premolars belonging to the same species for which proportions are respected.

We therefore attribute ND-2015-12-7 to the sivaladapid strepsirrhines because it possesses a combination of features that is only found in these Asian adapoids:

(1) The m1 displays an entoconid and a hypoconulid close to each other with a deep notch separating both cusps (Fig. 3), which are two diagnostic features of this family^31,34^. Although the hypoconulid is close to the midline of the tooth and not in lingual position as it is most typically the case in Neogene sivaladapids and some Paleogene representatives of the family, recently documented Paleogene Sivaladapidae greatly increase the known variability of this character. For instance, *Laomaki* and *Yunnanadapis* (Early Oligocene, China) display, as *Anthradapis* does, a hypoconulid close to the midline of the tooth appressed to the entoconid and separated from it by a deep notch^19^ (Fig. 3). Another variable feature among Paleogene Sivaladapids is the size of the hypoconulid. All Neogene sivaladapids and several Paleogene sivaladapids possess an enlarged hypoconulid on molars, this state of character being considered as a diagnostic feature of sivaladapids^34^. However, *Hoanghonius*^35^ has smaller hypoconulids and those of *Paukkaungia* are even smaller and barely individualized from the postcristid^36^. Therefore, the talonid features displayed by *Anthradapis* correspond to the morphological range of the Paleogene sivaladapids and include two diagnostic characters of this family.

(2) The m1 possesses a mesiodistally-oriented cristid obliqua. Although the orientation of this crest on m1 is markedly oblique in the Sivaladapinae and *Rencunius*, several Paleogene sivaladapids show a much less mesiolingually-oriented (*Wailekia*, *Hoanghonius*) or even a perfectly mesiodistally-oriented cristid obliqua (*Paukkaungia*, *Kyitchaungia*) similarly to *Anthradapis*^35–37^ (Fig. 3).

(3) Its permanent premolars (especially p3) are very similar to those of Paleogene sivaladapids such as *Paukkaungia*, *Guangxilemur*, *Laomaki* or *Yunnanadapis* (Fig. 5). The premolars of these taxa are convex buccally, have long trigonids bearing only a tall protoconid that is often mesially positioned, a complex crest pattern with several crests connected to the protoconid, a large talonid basin, and a short and low talonid. Their p3-p4 have four crests running mesially, distally, distolingually and distobuccally from the protoconid; the distolingual and distobuccal crests delimit the talonid basin; the distal postprotocristid projects in the middle of this basin and generally joins a short cristid obliqua descending from the hypoconid. The hypoconid is connected to a distocristid that closes the talonid basin distally^19,36,38^.

(4) The structure of its dp4 corresponds well to that of *Sivaladapis* (Fig. 4) which is elongate, well molarized, trilobate with a wide and lingually open trigonid basin surrounded by a long and markedly lingually curved preprotocristid.

Among sivaladapids, *Anthradapis* markedly differs from the Neogene Sivaladapinae, which possess molars with sharp crests, molarized p4, single-rooted, high, and caniniform/subcaniniform p2, three-crested protoconid on lower premolars (see also Supplementary Tables S2 and S3). Conversely, the Paleogene sivaladapids (Hoanghoniinae, Wailekiinae) are morphologically closer to *Anthradapis* in displaying a non-molarized p4, similar p2-p4 crown morphology and root pattern (Tables S2 and S3), and comparable tooth proportions for p3-p4 (Supplementary Table S1). However, none of these taxa possesses four premolars, non-reduced p1-p2 with the same complex crest pattern observed on p3-p4, comparable mandibular depth, molar crown height and occlusal outline, marked bunodonty, reduction of crests, opening and depth of the talonid basin, and accessory cuspules. In addition, *Anthradapis* differs from all Paleogene sivaladapids except *Kyitchaungia* and *Paukkaungia* by a distinct mesiodistal orientation of the m1 cristid obliqua. *Lushius* cannot be directly compared with *Anthradapis* since it is known by a partial maxilla. Nevertheless, this sivaladapid is considerably smaller than *Anthradapis* with estimated body weights of 1.45 kg^7^ and 2.3 kg^32^.

## Discussion

The entirely new combination of characters displayed by *Anthradapis* (see also Supplementary Tables S2 and S3) indicates that it belongs to a so far unsampled group of sivaladapids that has evolved in parallel with other Paleogene sivaladapids by developing marked bunodonty on molars, complex crest pattering on anterior premolars, but preserving plesiomorphic premolar formula and proportions. The retention in *Anthradapis* of numerous plesiomorphic traits found among Early Eocene adapoids including a p1 suggests that the branching of the Sivaladapidae among strepsirrhines is basal and much older than the Late Eocene. Indeed, Middle/Late Eocene adapoids (exclusive of adapids) do not typically possess a p1^21,31,32^.

The diversification of Eocene sivaladapids led several authors to investigate the phylogenetic relationships of this family among Paleogene strepsirrhines. The Middle Eocene cercamoniid *Periconodon* was hypothesized to represent a close relative of sivaladapids^30^. More recently, it has been suggested that the Early Eocene Asiadapidae were closely related to sivaladapids based on a phylogenetic analysis and a few dental features shared between *Marcgodinotius*, *Paukkaungia* and *Guangxilemur*^31^. These features include a transverse and deeply notched protocristid and a shallow notch between the hypoconid and the hypoconulid on the molars, a lingually positioned hypoconulid, a buccal cristid obliqua, similarities in the structure of dP4, and a simple p4 without paraconid or metaconid^31^. The last feature is regarded here as plesiomorphic and thus cannot support phylogenetic affinities between Asiadapidae and Sivaladapidae. It is also likely that the deep trigonid notch on the lower molars represents a plesiomorphic trait, other Paleogene sivaladapids showing generally higher protocristids. Some of the similarities noted in the structure of the dP4 between the Asiadapidae and *Guangxilemur* may also be plesiomorphic considering that the Asiadapidae possess a dp4 morphology close to that of *Donrussellia*^31^. Thus, the most reliable synapomorphies of *Marcgodinotius* and sivaladapids are those noted on the lower molars. Further comparisons between *Anthradapis* and the Asiadapidae suggest that *Asiadapis*, in contrast to *Marcgodinotius*, is already distant from the hypothetic ancestral morphotype of the sivaladapids in lacking a p1 and in having a single-rooted p2^31,39^. By displaying a buccally-positioned cristid obliqua and a metaconid and protoconid transversely in line, the morphology of *Anthradapis* suggests that these derived features may have been inherited early in the history of the sivaladapids given that this taxon belongs to a different group of sivaladapids than *Paukkaungia*. We note that this interpretation, which needs to be firmly demonstrated, does not contradict the hypothesis of close relationship between asiadapids and sivaladapids^31^. The dp4 of *Anthradapis* closely resembles those of *Asiadapis* and *Marcgodinotius*. Some of the shared trigonid features between these taxa (long mesial lobe with long and arcuate preprotocristid and wide trigonid basin) are also found in *Sivaladapis* but not in *Donrussellia*. These features can therefore be interpreted as apomorphic and they increase the number of shared derived features between Asiadapidae and Sivaladapidae. Thus, while some characters of *Anthradapis* exclude *Asiadapis* from the direct ancestry of the Sivaladapidae, the combination of features displayed by *Anthradapis* tends to reinforce the hypotheses of an early and basal origin of the sivaladapids among strepsirrhines and of a close relationship with the Asiadapidae. In order to test this hypothesis, and others related to sivaladapid relationships, we performed a phylogenetic analysis of strepsirrhines including *Anthradapis*.

**Figure 6:**
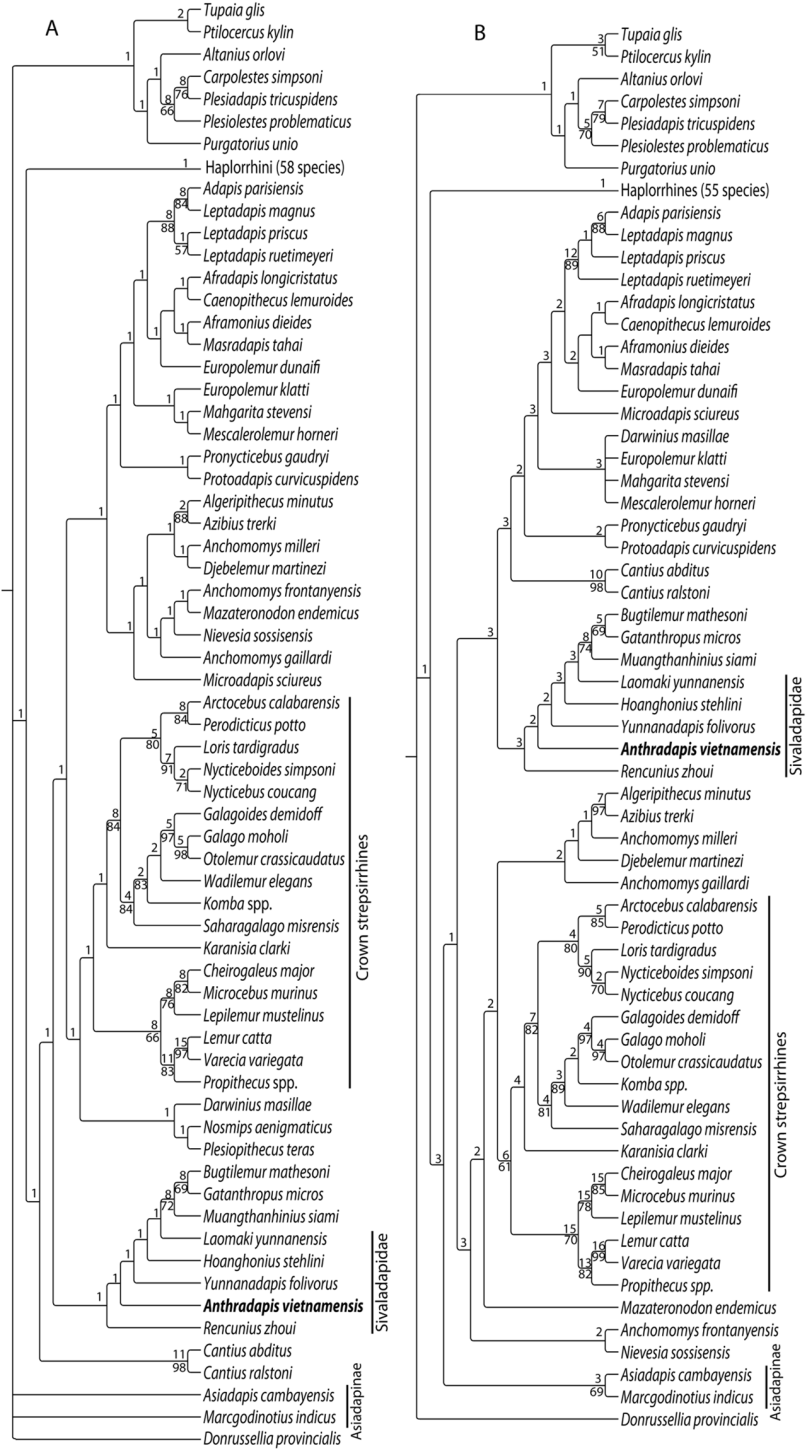
Phylogenetic position of *Anthradapis* among primates retrieved from two maximum parsimony analyses. (**A**) full taxonomic sampling (123 taxa). Strict consensus of 14 most-parsimonious trees (length = 4832.5, consistency index (CI) = 0.149, retention index (RI) = 0.574, rescaled consistency index (RC) = 0.085). (**B**) alternate taxonomic sampling (117 taxa) with 5 unstable taxa and the doubtful *Amphipithecus* discarded. Strict consensus of 9 most-parsimonious trees (length = 4700, CI = 0.153, RI = 0.577, RC = 0.088). The numbers above branches are Bremer support values. Bootstrap frequencies (>50) are indicated below branches.

Phylogenetic analyses based on a datamatrix used in a recent analysis of strepsirrhine phylogeny^28^ retrieve *Anthradapis* as a sivaladapid (Fig. 6; Methods). This taxon is nested in a clade that includes sivaladapids and Asian ekgmowechashalids (*Bugtilemur*, *Muangthanhinius*, *Gatanthropus*). The inner nodes of this clade, which do not group the two hoanghoniines *Rencunius* and *Hoanghonius* and are poorly supported, probably do not reflect reliable intra-sivaladapid relationships. The Sivaladapidae that are paraphyletic when Asian ekgmowechashalids are included in the analyses (contrary to another topology in which ekgmowechashalids and sivaladapids are sister-groups^19^), are adapoids with either a basal or a more nested placement within strepsirrhines. Contrary to previous results^31^, the Asiadapidae have a clearly more basal position than the sivaladapids in both analyses, which is in agreement with recent maximum parsimony analyses^7,28^, but not with Bayesian analyses^28^. Although the relative position of the Asiadapidae and the Sivaladapidae seems to be still unstable in recent phylogenies, our phylogenies do not support the hypothesis of a close relationship between these groups.

*Anthradapis* represents the largest known sivaladapid with an estimated bodyweight of 5.3 kg (see Methods). This body weight is slightly greater than those estimated for the formerly largest sivaladapids (*Sinoadapis*, Miocene of China, 4.7–4.4 kg*; Guangxilemur tongi*, Eocene of China, 4.8 kg^7^), and much higher than Kay’s^40^ threshold (500 g), over which primates obtain their protein from leaves instead of insects. Considering the very bunodont morphology of the molar with very reduced crests, a folivorous diet which is the common dietary inference for the Sivaladapinae and some Paleogene sivaladapids such as *Yunnanadapis*^8,19^, is unlikely for *Anthradapis*. Instead, a frugivorous diet can be hypothesized for *Anthradapis* based on its very bunodont m1 and its >5 kg body mass. Interestingly, the high-crowned and very bunodont m1 of *Anthradapis* recalls that of the parapithecid anthropoid *Qatrania* (body weight ≤500 g). This genus was interpreted as a frugivorous or gummivorous taxon based on its molar shearing quotient^41^. *Anthradapis* also possibly included hard items such as seeds in its diet, the combination of a strong canine, a deep mandibular corpus, and bunodont molars being found in durophagous anthropoids^42^. When using a platyrrhine model for m1 shearing quotients^41^ (strepsirrhine model only available for m2), we obtain value of −9.4 for *Anthradapis*. This value falls in the range of fruit/seed eaters among platyrrhines (−2.1 to −14.2) and is closer to those of specialized seed eaters such as *Cacajao* (−14.2) and *Chiropotes* (−11.2). Based on these various elements, a frugivorous diet including a significant proportion of seeds can be proposed for *Anthradapis*. The thin m1 enamel of *Anthradapis* is not typical of durophagy, this diet being often associated with thicker enamel^43^. If *Anthradapis* was consuming hard items, they were not processed by the molars but perhaps mostly by its large canines, like in *Chiropotes*, a pitheciin platyrrhine that uses its hypertrophied canines to break seeds and possesses molars with thin enamel^43^ (RET = ~8–10).

Among Eocene Asian primates, the Middle Eocene amphipithecid anthropoid *Ganlea* also shares with *Anthradapis* molar bunodonty with reduced crests, deep jaw and large canine. *Ganlea* was considered as a possible seed eater by comparison with pitheciins like *Chiropotes*^16^ and may have occupied the same dietary niche as *Anthradapis*.

## Conclusions

The discovery of the medium-sized primate *Anthradapis* represents the first record of an Eocene primate in Vietnam. This discovery extends the body mass range of the mammalian fossil community in the Na Duong locality and demonstrates that its mammalian biodiversity is still poorly known. The unique combination of features displayed by *Anthradapis* indicates that it belongs to a new subfamily of sivaladapids, the Anthradapinae, which has evolved in parallel with other Paleogene sivaladapids such as the Hoanghoniinae, and therefore reveals a completely new part of sivaladapid evolution. The retention of a p1 and non-reduced anterior premolars in *Anthradapis* refines our knowledge of the ancestral morphotype of the sivaladapids and suggests a rather ancient and basal branching of the family within the strepsirrhines. *Anthradapis* adds a few presumed dental synapomorphies between the Sivaladapidae and the Asiadapidae, which appears to reinforce the hypothesis of a close relationship between these groups of primates. However, this result is not confirmed by our phylogenetic analyses, perhaps because the morphological gap between the Paleogene Sivaladapidae and the Asiadapidae is still quite large. More complete remains or even additional basal representatives of the sivaladapids, in particular primitive Anthradapinae, will be necessary to further constrain the phylogenetic position of the sivaladapids and the morphological characteristics of their early representatives.

## Methods

### X-ray microtomography

The specimen was scanned using an EasyTom HR-microtomograph with a voxel size of 17.49 µm. Scan parameters: X-ray voltage = 65 kV, current = 270 uA, number of projections = 2880, filter = Tukey, framerate = 4 frame s^−1^.

### Phylogenetic analysis

The phylogenetic position of *Anthradapis* among primates was retrieved with a maximum parsimony analyses in PAUP 4.0b10^44^ based on the data matrix of recent phylogenetic analysis of strepsirrhine primates^28^ (122 taxa and 394 characters) augmented with *Anthradapis*. Most-parsimonious trees were obtained following heuristic searches with 1000 replications and random addition of taxa. Two different analyses were performed: one with the full taxonomic sample (123 taxa). A second analysis was performed after removing five unstable taxa (*Afrotarsius* spp., *Afrasia djijidae*, *Rooneyia viejaensis*, *Nosmips aegnimaticus* and *Plesiopithecus teras*) and the doubtful taxon *Amphipithecus mogaungensis* considered here as synonymous with *Pondaungia cotteri*^45^. Recovered topologies have been constrained in both analyses with a backbone tree^28^. The datamatrix and the constraint tree used for these analyses are available as Supplementary Data 1 and 2.

### Relative enamel thickness

The 2D relative enamel thickness^46^ (RET) of *Anthradapis vietnamensis* was estimated on the little worn m1 of ND-2015-12-7. This methodology has been preferred over 3D RET because the contrast between enamel and dentine did not allow a clear separation between these tissues on the whole tooth. We have determined the RET along a hypoconid/protoconid section (RET = 5.89) and a hypoconid/entoconid section (RET = 6.17). The mean RET based on these two sections is 6.02.

### Body weight

The body weight of *Anthradapis vietnamensis* was estimated using the regression equation for prosimians^47^ between the surface area (S) of the m1 (mesiodistal length × buccolingual breadth) and the body weight (BW) applied on the m1 of the holotype: $\mathrm{ln}\,BW=1.614389\ast \,\mathrm{ln}\,S+2.666647.$

## Supplementary information

Supplemental information

Supplemental information 2

Supplemental information 3

Supplemental information 4

Supplemental information 5

Supplemental information 6

Supplemental information 7
