# Supplementary material for: A new primate from the late Eocene of Vietnam illuminates unexpected strepsirrhine diversity and evolution in Southeast Asia
Source: Sci Rep. 2019 Dec 27;9:19983. doi: 10.1038/s41598-019-56255-8 (PMC6934687; doi:10.1038/s41598-019-56255-8)
Supplement: Supplementary file 1 — Supplemental information [file 41598_2019_56255_MOESM1_ESM.pdf]

Supplementary material for

A new sivaladapid primate from the late Eocene of Vietnam reinforces the basal branching of the Sivaladapidae among strepsirrhines.

Olivier Chavasseau, Yaowalak Chaimanee, Stéphane Ducrocq, Vincent Lazzari, Phan Dong Pha, Mana Rugbumrung, Jérôme Surault, Dang Minh Tuan, and Jean-Jacques Jaeger.

Comparisons with non-primate taxa

Because of its very peculiar morphology, the ordinal identification of ND-2015-12-7 has necessitated comparisons with several orders of eutherian mammals. The Holarctic early Cenozoic 'condylarthrans' Phenacodontidae possess bunodont lower molars with entoconid and hypoconulid spaced closely and strongly molarized dp4. However, none of them match well the Vietnamese mandible from a morphological point of view: these mammals possess strong and oblique cristid obliqua, reduced paraconid and lophodont pattern (*Ectocion*, *Lophocion*) on lower molars, broader dp4 with more compressed mesial lobe, stronger and more oblique cristid obliqua, submolariform p4, more symmetrical anterior premolars, longer jaws with diastema between premolars (see ref[1] for genera *Phenacodus*, *Tetraclaenodon* and *Ectocion* and ref[2] for genera *Palasiodon*, *Yuodon and Lophocion*). The temporal and geographical distributions of the phenacodontids also do not correspond to those of *Anthradapis* since they are rare in Asia with a temporal range limited to the Early Eocene[2-4]. According to a review of Asian condylarths[2], only two other condylarth families are represented in the Paleogene of Asia, exclusively during the Early Eocene: the Hyopsodontidae and the Quettacyonidae. The Hyopsodontidae possess more molarized and transverse premolars and m1/m2 without paraconid, more open talonid basins and sometimes marked lophodonty (e.g, refs[2,5] for genera *Hypsodus* and *Midiagnus*). The Quettacyonidae (genus *Quettacyon*) differ markedly from *Anthradapis* in having enlarged, rectangular premolars with inflated protoconids and a more simple crest pattern[2].

Another investigated group was the cetartiodactyls with basal representatives that possess bunodont molars and trilobed dp4. In the reassessement to the cetartiodactyls of the Eocene genus *Lantianus*, formely thought to belong to the primates, the length of the snout was a key feature to distingh primates from cetartiodactyls, the latter being characterized by much longer snouts[6]. Owing to its short jaw, *Anthrapis* possessed a short snout, which excludes it

from the cetartiodactyls. Moreover, the combination observed in *Anthradapis* of a short lower jaw with upwardly oriented incisors, large canines, short, tall and complex premolars not separated by diastema, shortened and bunodont lower molars with a mesially oriented cristid obliqua, a centroconid and a well-developed paraconid not appressed to the metaconid is unknown in primitive cetartiodactyls[7-9].

Phylogenetic analysis at the scale of eutherian mammals

We have used the datamatrix of ref[10] to test the phylogenetic position of *Anthradapis* among eutherian mammals (70 taxa, 408 characters). Although only ~40 characters could be coded for *Anthradapis*, our analyses invariably place this genus within the euarchontoglires, ruling out phylogenetic affinities of *Anthradapis* with condylarths or 'ungulates' (Fig. S1). We note that our morphological analysis of *Anthradapis* did not enlighten any typical glire synapomorphy in this fossil.

Contact facets are present on the distal wall of the m1 on ND-2015-12-7. This demonstrate that *Anthradapis* possessed at least 6 postcanine teeth. When coding the feature 3 (number of postcanine teeth) for *Anthradapis*, adding the very reasonable assumption that *Anthradapis* possessed 7 postcanine teeth (state 1), the loss of m3 being rare in eutherian mammals outside of glires, carnivores and creodont, *Anthradapis* groups with primates in the majority-rule consensus tree (Fig. S2). The datamatrix used for this analysis is available as a Supplementary Data 3.

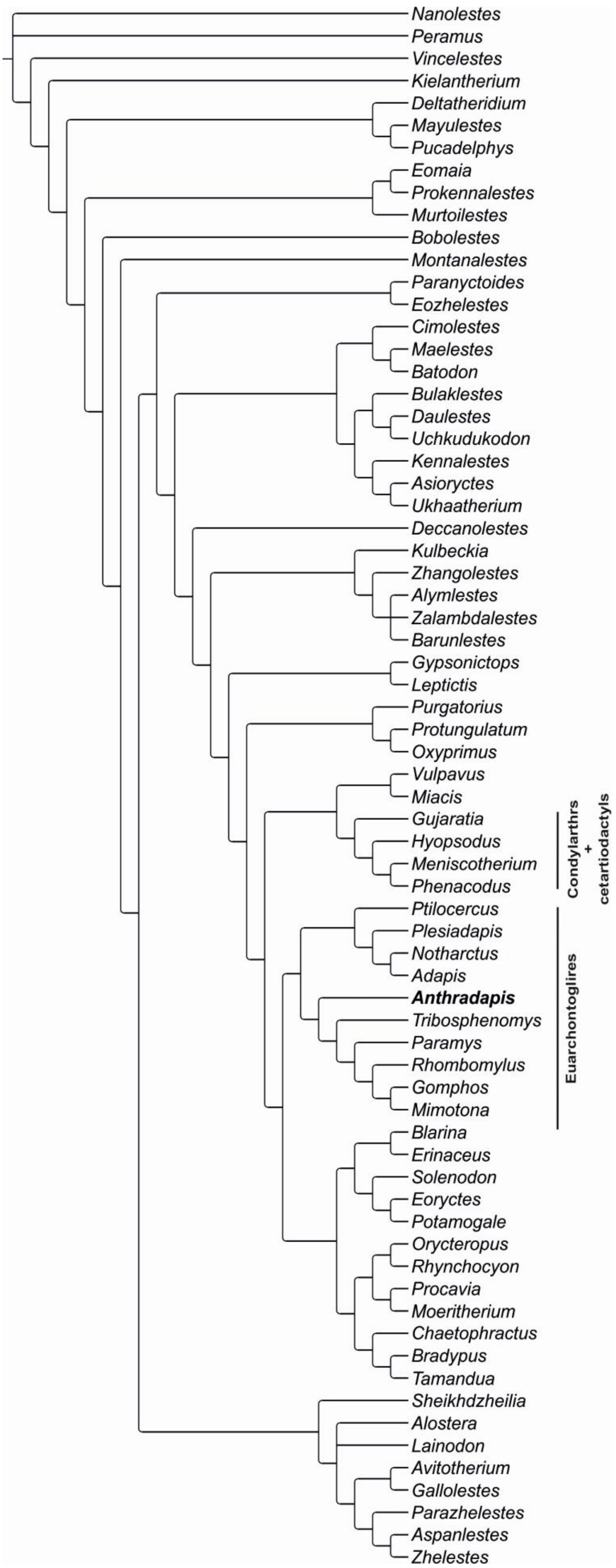

Figure S1. Retrieved phylogenetic position of *Anthradapis* among eutherian mammals. Strict consensus of 9 most-parsimonious trees of 2303 steps obtained with a heuristic search in Paup 4.0b10 (random addition of taxa, 500 replications of heuristic search). CI=0.2601, RI=0.5508, RC=0.1432.

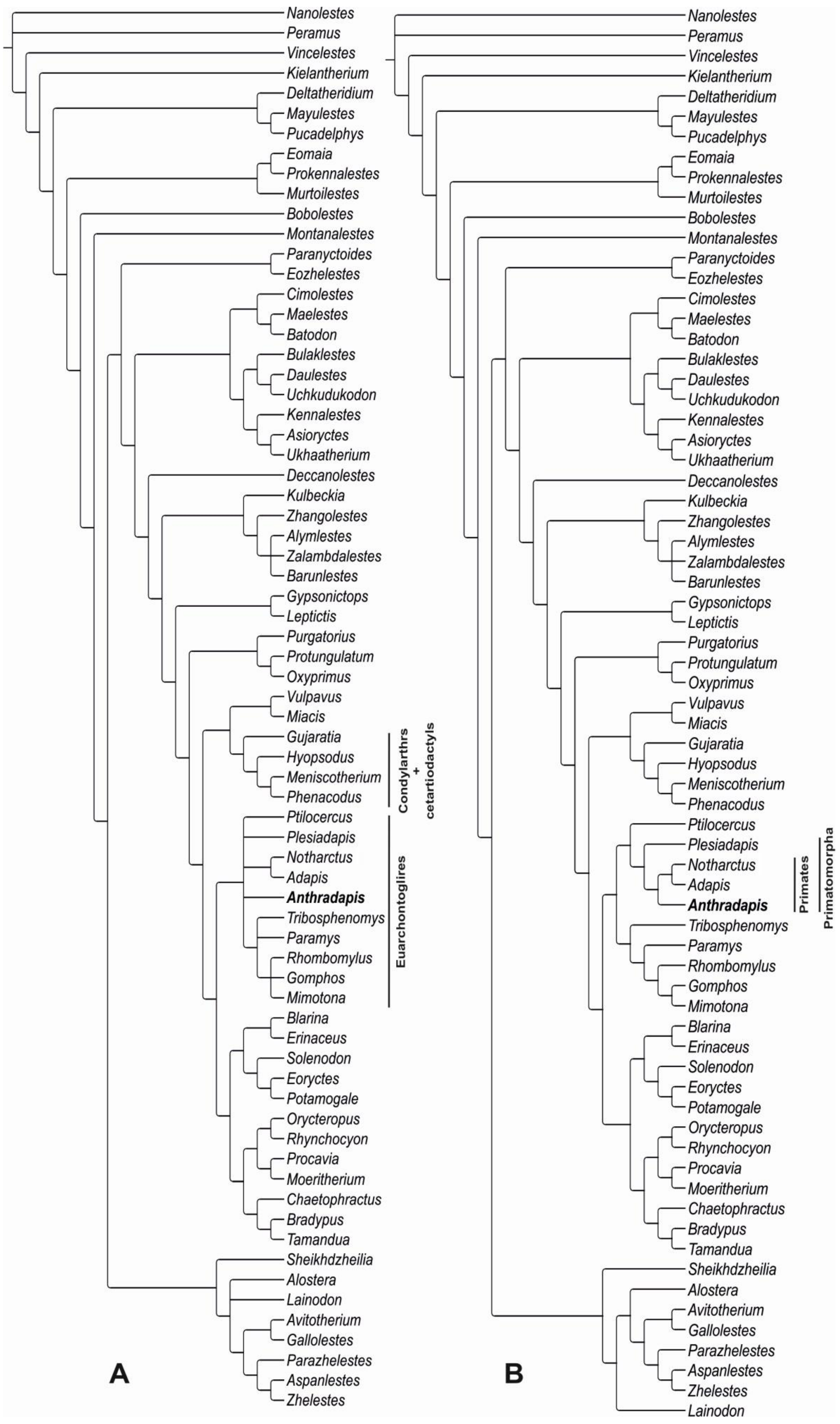

Figure S2. Retrieved phylogenetic position of *Anthradapis* among eutherian mammals. A: strict consensus of of 312 most-parsimonious trees of 2304 steps obtained with a heuristic search in Paup 4.0b10 (random addition of taxa, 500 replications of heuristic search). CI=0.26 RI=0.55 RC=0.14. B: Majority-rule consensus tree. Frequency of the *Notharctus*+*Adapis*+*Anthradapis* clade in the most-parsimonious trees= 87%.

Supplementary tables captions

Table S1. Metrical comparisons of dento-gnathic characters of *Anthradapis* with other sivaladapids.

Table S2. Comparison of mandibular and premolar features between *Anthradapis* and Asian adapoids (sivalalapids and asiadapines). Legend for the characters of *Anthradapis*: character in normal font = commonly found among sivaladapids or Asian adapoids; underlined character = feature found among Asian adapoids but less commonly; character in bold = autapomorphy of *Anthradapis*. Abbreviations: E. = Early; M. = Middle; L. = Late; Eoc. = Eocene; Oligoc. = Oligocene; Mioc. = Miocene.

Table S3. Comparison of molar features between *Anthradapis* and Asian adapoids (sivalalapids and asiadapines). Legend for the characters of *Anthradapis* and abbreviations identical to those of table 2.

References

1 Thewissen, J. G. M. Evolution of Paleocene and Eocene Phenacodontidae (Mammalia, Condylarthra). *Univ. Mich. Pap. Paleontol.* **29**, 1-107 (1990).

2 Kondrashov, P. E. & Lucas, S. G. Revised distribution of condylarths (Mammalia, Eutheria) in Asia. *Bull. N. M. Nat. Hist. Sci.* **26**, 209-214 (2004).

3 Rose, K. D. *The Beginning of the Age of Mammals*. (The Johns Hopkins University Press, 2006).

4 Russell, D. E. & Zhai, R.-j. The Paleogene of Asia: mammals and stratigraphy. *Mem. Mus. Natl. Hist. Nat. Sér. C* **52**, 1-488 (1987).

5 Flynn, J. J. *Hyopsodus* (Mammalia) from the Tepee Trail Formation (Eocene), Northwestern Wyoming. *Am. Mus. Novit.* **3007**, 1-19 (1991).

6 Gingerich, P. D. Systematic position of the alleged primate *Lantianius xiehuensis* Chow, 1964, from the Eocene of China. *J. Mammal.* **57**, 194-198 (1976).

7 Hooker, J. J. & Thomas, K. M. A new species of *Amphirhagatherium* (Choeropotamidae, Artiodactyla, Mammalia) from the Late Eocene Headon Hill Formation of southern England and phylogeny of endemic European `Anthracotherioids'. *Palaeontology* **44**, 827-853 (2001).

8 Kondrashov, P. E., Lopatin, A. V. & Lucas, S. G. The oldest known asian artiodactyl (Mammalia). *Bull. N. M. Nat. Hist. Sci.* **26**, 205-208 (2004).

9 Theodor, J. M., Erfurt, J. & Métais, G. in *The Evolution of Artiodactyls* (eds Donald R. Prothero & S. E. Foss) 32-58 (The Johns Hopkins University Press, 2007).

10 Wible, J. R., Rougier, G. W., Novacek, M. J. & Asher, R. J. Cretaceous eutherians and Laurasian origin for placental mammals near the K/T boundary. *Nature* **447**, 1003-1006 (2007).
