# Supplementary material for: A new primate from the late Eocene of Vietnam illuminates unexpected strepsirrhine diversity and evolution in Southeast Asia
Source: Sci Rep. 2019 Dec 27;9:19983. doi: 10.1038/s41598-019-56255-8 (PMC6934687; doi:10.1038/s41598-019-56255-8)
Supplement: Supplementary file 5 — Supplemental information 5 [file 41598_2019_56255_MOESM5_ESM.docx]

| **Taxon** | **Specimen number** | | **p2:p3  occlusal area^a^** | **(MD p2+ MD p3+**  **MD p4): MD m1** | **(MD p3+**  **MD p4) : MD m1** | **MD p4: MD m1** | **MD: BL m1** | **Mand. depth  at m1: MD m1** | | **Reference** | | |
| --- | --- | --- | --- | --- | --- | --- | --- | --- | --- | --- | --- | --- |
| ***Anthradapis vietnamensis*** | | **ND-2015-12-7** | **0.76** | **>2.29** | **>1.64** | **>0.84** | **1.15** | **~2.00** | | **This study** | | |
| ***Ramadapis sahni*** | | **VPL/RSP1** | **-** | **-** | **-** | **1.18** | **1.38** | **1.81** | | **Gilbert et al. (2017)** | | |
| *Sivaladapis palaeindicus* | | YGSP 32154 | - | - | - | - | 1.30 | - | | Flynn and Morgan (2005) | | |
| *Sivaladapis palaeindicus* | | YGSP 46458 | - | - | - | - | 1.33 | 1.81 | | Flynn and Morgan (2005) | | |
| *Sivaladapis palaeindicus* | | unnumbered | - | - | - | 1.06 | 1.36 |  | | Thomas and Verma (1979) | | |
| ***Sivaladapis palaeindicus* mean** | |  | **-** | **-** | **-** | **1.06** | **1.33** | **1.81** | | **-** | | |
| *Sivaladapis nagrii* | | LUVP 14500/116860 | - | - | - | 0.99 | 1.45 | - | | Gilbert et al. (2017) | | |
| *Sivaladapis nagrii* | | GSI 18093 | - | - | - | - | 1.23 | - | | Gilbert et al. (2017) | | |
| *Sivaladapis nagrii* | | PUA 826-69 | - | - | - | 1.02 | 1.26 | - | | Gilbert et al. (2017) | | |
| *Sivaladapis nagrii* | | PUA 72-10 | - | - | - |  | 1.17 |  | | Gilbert et al. (2017) | | |
| *Sivaladapis nagrii* | | PUA 736-69 | - | - | - | 1.11 | 1.26 |  | | Gilbert et al. (2017) | | |
| ***Sivaladapis nagrii* mean** | |  | **0.72** | **2.77^b^** | **1.99^b^** | **1.04** | **1.27** | **1.97^c^** | | **Gingerich and Sahni (1984)** | | |
| ***Indraloris himalayensis*** | | **YPM 13802** | **-** | **-** | **-** |  | **1.21** |  | | **Gilbert et al. (2017)** | | |
| ***Indraloris kamlialensis*** | | **YGSP 44443^d^** | **-** | **-** | **-** |  | **>1.2** | **-** | | **Flynn and Morgan (2005)** | | |
| ***Indraloris* large sp.** | | **YGSP 32152** | **-** | **-** | **-** | **-** | **1.18** | **-** | | **Flynn and Morgan (2005)** | | |
| ***Sinoadapis carnosus*** | | **PA 885** | **-** | **-** | **-** | **1.19** | **1.19^e^** | **2.63** | | **Pan and Wu (1986)** | | |
| ***Sinoadapis shihuibaensis*** | | **PA 882** | **0.75** | **2.82** | **2.04** | **1.19** | **1.28** | **2.51** | | **Pan and Wu (1986)** | | |
| *Siamodapis maemohensis* | | TF 6273 | - |  |  | 1.04 | 1.46 | 1.94 | | Chaimanee et al. (2008) | | |
| *Siamodapis maemohensis* | | TF 6233 | - |  |  | 1.14 | 1.39 | 1.87 | | Chaimanee et al. (2008) | | |
| ***Siamodapis maemohensis* mean** | |  | **-** |  |  | **1.09** | **1.43** | **1.91** | | **-** | | |
| **Range/Mean for Neogene sivaladapids^f^** | |  | **0.72-0.75/0.74** | **2.77-2.82/2.80** | **1.99-2.04/2.02** | **1.04-1.19/1.13** | **1.18-1.43/1.27** | **1.81-2.63/2.11** | | - | | |
| *Hoanghonius stehlini* | | IVPP V10220 | 0.52 | 2.18 | 1.64 | 0.87 | 1.39 | 1.81^g^ | | Tong et al. (1999) | | |
| *Guangxilemur singsilai* | | DBC 2170 | - | - | - | - | 1.45 | - | | Marivaux et al. (2002) | | |
| *Guangxilemur singsilai* | | DBC 2171 | - | - | - | - | 1.39 | - | | Marivaux et al. (2002) | | |
| *Guangxilemur singsilai* mean | |  | - | - | - | - | 1.42 | - | | - | | |
| *Rencunius zhoui* | | IVPP 5312 | - | - | - | 0.95 | 1.30 | - | | Gingerich et al. (1994) | | |
| *Rencunius zhoui* | | IVPP 5311 | - | - | - | - | 1.45 | - | | Woo and Chow (1957) | | |
| *Rencunius zhoui* mean | |  | - | - | - | 0.95 | 1.37 | - | | - | | |
| *Paukkaungia parva*^h^ | |  | - | - | 1.69 | 0.99 | 1.47 | - | | Beard et al. (2007) | | |
| *Laomaki yunnanensis*^i^ | |  | - | - | 1.70 | 0.88 | 1.29 | - | | Ni et al. (2016) | | |
| *Yunnanadapis folivorus* | | IVPP V 22702 | 0.42 | 2.29 | 1.77 | 0.94 | 1.40 | - | | Ni et al. (2016) | | |
| **Range/Mean for Paleogene sivaladapids^f^** | |  | **0.42-0.52/0.47** | **2.18-2.29/2.23** | **1.64-1.77/1.70** | **0.87-0.99/0.93** | **1.29-1.47/1.39** | **1.81** | |  | | |
|  | |  |  |  |  |  | | |  | |  |  |

^a^occlusal area computed as MDxBL ^b^ calculated with mean p2-m1 measurements given by Gingerich and Sahni (1984) for *Sivaladapis nagrii* ^c^ Ratio of mean mandibular depth to mean m1 mesiodistal length calculated with data given by Gingerich and Sahni (1984)  ^d^ m1 or m2 according to Flynn and Morgan (2005). This tooth was considered here as a m1.  ^e^ mean of left and right m1 ratios ^f^ Calculated on species mean values ^g^ measured from figure.
^h^calculated with NMMP 54 (isolated p3), NMMP 56 (isolated p4) and NMMP 55 (holotype isolated m1)
^i^calculated with isolated teeth IVVP V 22709 (p3), IVPP V 22710 (p4) and IVPP V 22711 (m1)
