# Supplementary material for: A new primate from the late Eocene of Vietnam illuminates unexpected strepsirrhine diversity and evolution in Southeast Asia
Source: Sci Rep. 2019 Dec 27;9:19983. doi: 10.1038/s41598-019-56255-8 (PMC6934687; doi:10.1038/s41598-019-56255-8)
Supplement: Supplementary file 6 — Supplemental information 6 [file 41598_2019_56255_MOESM6_ESM.pdf]

Character/Taxon	*Anthradapis* L. Eoc. Vietnam	*Hoanghonius* M. Eoc. China	*Laomaki* E. Oligoc. China	*Yunnanadapis* E. Oligoc. China	*Kyitchaungia* M. Eoc. Myanmar	*Paukkaungia* M. Eoc. Myanmar	*Rencunius* M. Eoc. China	*Guangxi-lemur* E. Oligoc. Pakistan	*Wailekia* L. Eoc. Thailand	*Ramadapis* M. Mioc. India	*Sinoadapis* L. Mioc. China	*Indraloris* M./L. Mioc. Pakistan India	*Sivaladapis nagrii* L. Mioc. India	*Siamoadapis* M. Mioc. Thailand	*Marcgodinotius* E. Eoc. India	*Asiadapis* E. Eoc. India
Corpus relative depth	Moderate	Low	Low?	?	?	?	Moderate?	?	Moderate?	Low	High	Low	Moderate	Low	Low	Low
Canine crown height	High	High	?	Moderate	?	?	?	?	?	?	High	?	High	?	High	?
Size of canine crown	Large	Large	?	Moderate	?	?	?	?	?	?	Large	Reduced	Large	Large	Large	?
Size of canine root	Massive	Massive	?	Massive	?	?	?	?	?	?	Massive	?	Massive	?	Massive	Massive?
Canine implantation	Subvertical	Subvertical	?	Subvertical	?	?	?	?	?	?	Subvertical	?	Subvertical	Subvertical	Subvertical	Subvertical?
Canine root distal curvature	**Important**	Slight	?	Absent	?	?	?	?	?	?	Slight	?	Slight	?	Slight	?
Canine crown cross-section	**Subtriangular** laterally compressed	Oval, laterally compressed	?	Oval, laterally compressed	?	?	?	?	?	?	Round	?	Oval, laterally compressed	?	Round	?
p1	Present	Absent	?	Absent	?	?	?	?	Absent	?	Absent	Absent	Absent	Absent	Present	Absent
p2 root number	2	2	?	2	?	?	?	?	2	?	1	?	1	1	2	1
Relative height of p2 to p3	**p2≈p3**	p2<p3	?	p2<< p3	?	?	?	?	?	?	p2>p3	?	p2>>p3	?	?	?
p2 crown pattern	Premolariform	Premolariform	?	Premolariform	?	?	?	?	?	?	Subcaniniform	?	Caniniform	?	?	?
Size of p2 relative to p3	Large p2	Reduced p2		Reduced p2	?	?	?	?	?	?	Large p2	?	Large p2	?	?	?
Number of crests starting from protoconid on p2/p3/p4	**4**/4/4?	2/4?/4?	?/4/4	4/4/4	?	?/3/4	?/?/4	?	?	?	2/4/3	?/3/3	2/3/3	?/3/3	?/2/3	?/2/3
Relative height of p3 to p4	p4>p3	p3> p4	p4>p3	p3>>p4	?	p4>p3	?	?	?	?	p3> p4	p3> p4	p3> p4	?	p3> p4	p3> p4
Length of p4 relative to m1	p4<m1	p4<m1	p4<m1	p4≈m1	?	p4≈m1	p4≈m1	?	?	p4>m1	p4>m1	p4>m1	p4>m1	p4>m1	p4≈m1	p4≈m1
p4 metaconid	Absent?	Small	Small	Large	?	Absent	Small	?	?	?	Large	Large	Large	Large	Absent	Abs./Small
Crown pattern of p4	Premolariform	Premolariform	Premolari-form	Submolari-form	?	Premolari-form	Premolari-form	?	?	?	Submolari-form	Submolari-form	Submolari-form	Submolari-form	Premolari-form	Premolari-form
p4 hypoconid	Small	Small	Small	Large	?	Small	Small	?	?	Large	Large	Large	Large	Large	Small	Small
Premolar talonid basins	Large	Large	Large	Large	?	Large	Large	Large	?	Large	Large	Large	Large	Large	Small	Small
Premolar preprotocrista	Straight	Straight	Curved lingually	Curved lingually	?	Straight	Straight	Straight	?	?	Curved lingually	Curved lingually	Curved lingually	Curved lingually	Straight	Straight
Premolar paraconid	Absent	Absent	Absent	Absent	?	Absent	Absent	Absent	?	?	Absent	Absent	Absent	Absent	Absent	Absent
dp4 shape	Elongate, trilobate	?	?	?	?	?	?	?	?	?	?	Elongate, trilobate[a]	Elongate, trilobate	?	Elongate, trilobate	Elongate, trilobate
dp4 trigonid	Wide, open	?	?	?	?	?	?	?	?	?	?	Wide, open	Wide, open	?	Wide, open	Wide, open
dp4 preprotocristid	Long, curved	?	?	?	?	?	?	?	?	?	?	Long, curved	Long, curved	?	Long, curved	Long, curved
dp4 distal metaconid shift	Weak	?	?	?	?	?	?	?	?	?	?	Important	Weak	?	Important	Important
dp4 cristid obliqua	**Weak, mesial**	?	?	?	?	?	?	?	?	?	?	Strong, mesiolingual	Strong, mesiolingual	?	Strong, mesiolingual	Strong, mesiolingual
dp4 hypoconulid position	Closer to hypoconid	?	?	?	?	?	?	?	?	?	?	Lingual	Close to entoconid	?	Central/Closer to entoconid	Closer to hypoconid

[a]We consider that YGSP 24338 is a dp4 of *Indraloris kamlialensis* erroneously identified as a p4 by Flynn and Morgan (2005), this tooth being, with the exception of the lack of an entoconid, extremely similar to the dp4 of *Sivaladapis nagrii* in terms of shape and crest pattern
