# Supplementary material for: A new primate from the late Eocene of Vietnam illuminates unexpected strepsirrhine diversity and evolution in Southeast Asia
Source: Sci Rep. 2019 Dec 27;9:19983. doi: 10.1038/s41598-019-56255-8 (PMC6934687; doi:10.1038/s41598-019-56255-8)
Supplement: Supplementary file 7 — Supplemental information 7 [file 41598_2019_56255_MOESM7_ESM.pdf]

Character/Taxon	*Anthradapis*<br>L. Eoc.<br>Vietnam	*Hoanghonius*<br>M. Eoc.<br>China	*Laomaki*<br>E. Oligoc.<br>China	*Yunnanadapis*<br>E. Oligoc.<br>China	*Kyitchaungia*<br>M. Eoc.<br>Myanmar	*Paukkaungia*<br>M. Eoc.<br>Myanmar	*Rencunius*<br>M. Eoc.<br>China	*Guangxi-lemur*<br>E. Oligoc.<br>Pakistan	*Wailekia*<br>L. Eoc.<br>Thailand	*Ramadapis*<br>M. Mioc.<br>India	*Sinoadapis*<br>L. Mioc.<br>China	*Indraloris*<br>M./L. Mioc.<br>Pakistan<br>India	*Sivaladapis nagrii*<br>L. Mioc.<br>India	*Siamoadapis*<br>M. Mioc.<br>Thailand	*Marcgodinotius*<br>E. Eoc.<br>India	*Asiadapis*<br>E. Eoc.<br>India
m1 paraconid	Large	Small	Very small	Absent	?	Small	Large	Abs./very small	?	?	Absent	Absent	Small	Very small	Moderate/Large	Large
m1 trigonid mesiodistal length	Short	Long	Long	Long	?	Long	Long	Short	?	Short	Short	Short	Short	Long	Long	Long
Lower molar trigonid	Closed	Opened	Closed	Closed	?	Opened	Opened	Closed	?	Closed	Closed	Closed	Closed	Opened	Opened	Opened
Molar pattern/crest development	**Markedly bunodont, highly reduced crests**	Developed crests	Developed crests	Developed crests	Developed crests	Developed crests	Bunodont with reduced crests	Developed crests	Developed crests	Strongly developed crests	Reduced/ developed crests	Strongly developed crests	Strongly developed crests	Strongly developed crests	Developed crests	Developed crests
Molar crown height	High	Low	Low	low	Low	Low	Low	?	Low	High?	Moderate	High	High	Low	Low	Low
m1/m2 cristid obliqua	**Weak and short**	Strong	Strong	Strong	Strong	Strong	Strong	Strong	Strong	Strong	Strong	Strong	Strong	Strong	Strong	Strong
m1/m2 cristid obliqua direction	Mesial	Mesiolingual	Mesiolingual	Mesiolingual	Mesial	Mesial	Mesiolingual	Mesiolingual	Mesiolingual	Mesiolingual	Mesiolingua l	Mesiolingual	Mesiolingual	Mesiolingual	Mesial	Mesial/ Mesiolingual
m1/m2 cristid obliqua terminus	Distal to protoconid	Distal to protoconid	Distal to protoconid	Between protoconid & metaconid	Distal to protoconid	Distal to protoconid	Between protoconid & metaconid	Distal to protoconid	Distal to protoconid	Distal to protoconid	Distal to protoconid/ Between protoconid & metaconid	Distal to protoconid	Between protoconid & metaconid	Distal to protoconid	Distal to protoconid	Distal to protoconid
m1/m2 hypoconulid position relative to talonid cusps	Close to entoconid	Close to entoconid	Close to entoconid	Close to entoconid	Close to entoconid	Close to entoconid	Close to entoconid	Close to entoconid	Close to entoconid	Close to entoconid	Close to entoconid	Close to entoconid	Close to entoconid	Close to entoconid	Equally spaced between talonid cusps/Closer to entoconid	Equally spaced between talonid cusps/Closer to entoconid
Hypoconulid position	Close to midline	Lingual	Close to midline	Close to midline	Close to midline	Lingual	Lingual/Close to midline	Lingual	Lingual	Lingual	Lingual	Lingual	Lingual	Lingual	Central/Lingual	Central/Lingual
Notch between m1/m2 hypoconulid and entoconid	Deep	Deep	Deep	Deep	Deep	Shallow	Deep	Deep	Deep	Absent?	Deep	Deep	Deep	Deep	Absent	Absent
Size of m1/m2 hypoconulid	Moderate	Moderate	Large	Large	Large	Small/absent	Moderate/ Large	Large	Moderate	Large	Large	Large	Large	Large	Small	Small
Distal shift of metaconid on m1/m2	Weak	Strong	Strong	Weak	Strong	Weak	Strong	Strong	Weak	Weak	Weak	Strong	Strong	Strong	Weak	Weak
Shape of m1/m2	Squared	Rectangular	Rectangular	Rectangular	Rectangular	Rectangular	Rectangular	Rectangular	Rectangular	Rectangular	Rectangular/ Squared	Rectangular/ Squared	Rectangular	Rectangular	Rectangular	Rectangular
Centroconid	**Present**	Absent	Absent	Absent	Absent	Absent	Absent	Absent	Absent	Absent	Absent	Absent	Absent	Absent	Absent	Absent
m1/m2 talonid basin depth	Very deep	Deep	Very deep	Deep	Moderately deep	Deep	Deep	Deep	Deep	Very deep?	Deep	Very deep	Very deep	Deep	Deep	Deep
Hypocristid on m1 and m2	**Absent**	Present	Present	Present	Present	Present	Present but weak	Present	Present	Present	Present	Present	Present	Present	Present	Present
Distal wall of m1/m2 trigonid	Markedly notched	Weakly notched	Weakly notched	Weakly notched	Weakly notched	Markedly notched	Markedly notched	Weakly notched	Weakly notched	High	Weakly notched/high	High	High	High	Markedly notched	Markedly notched
m1/m2 buccal cingulum	**Absent**	Complete	Complete	Complete	?	Complete/ subcomplete	Complete	Complete/ subcomplete	Incomplete	Incomplete	Complete	Incomplete	Complete	Incomplete	Subcomplete	Subcomplete
